# Interactions between Gut Microbiota and Polyphenols: New Insights into the Treatment of Fatigue

**DOI:** 10.3390/molecules27217377

**Published:** 2022-10-30

**Authors:** Chuanhong Luo, Xichuan Wei, Jiao Song, Xiaorong Xu, Haozhou Huang, Sanhu Fan, Dingkun Zhang, Li Han, Junzhi Lin

**Affiliations:** 1 State Key Laboratory of Southwestern Chinese Medicine Resources, Pharmacy School, Chengdu University of Traditional Chinese Medicine, Chengdu 611137, China; 2 College of Nuclear Technology and Automation Engineering, Chengdu University of Technology, Chengdu 610051, China; 3 College of Acupuncture and Tuina, Chengdu University of Traditional Chinese Medicine, Chengdu 611137, China; 4 Innovative Institute of Chinese Medicine and Pharmacy, Chengdu University of Traditional Chinese Medicine, Chengdu 611137, China; 5 Sichuan Huamei Pharmaceutical Co., Ltd., Sanajon Pharmaceutical Group, Chengdu 610045, China; 6 TCM Regulating Metabolic Diseases Key Laboratory of Sichuan Province, Hospital of Chengdu University of Traditional Chinese Medicine, Chengdu 610072, China

**Keywords:** polyphenols, gut microbiota, fatigue, urolithin, interaction

## Abstract

Fatigue seriously affects people’s work efficiency and quality of life and has become a common health problem in modern societies around the world. The pathophysiology of fatigue is complex and not fully clear. To some degree, interactions between gut microbiota and host may be the cause of fatigue progression. Polyphenols such as tannin, tea polyphenols, curcumin, and soybean isoflavones relieve fatigue significantly. Studies have shown that the gut microbiota is able to convert these active compounds into more active metabolites through intestinal fermentation. However, the mechanism of anti-fatigue polyphenols is currently mainly analyzed from the perspective of antioxidant and anti-inflammatory effects, and changes in gut microbiota are rarely considered. This review focuses on gut microecology and systematically summarizes the latest theoretical and research findings on the interaction of gut microbiota, fatigue, and polyphenols. First, we outline the relationship between gut microbiota and fatigue, including changes in the gut microbiota during fatigue and how they interact with the host. Next, we describe the interactions between the gut microbiota and polyphenols in fatigue treatment (regulation of the gut microbiota by polyphenols and metabolism of polyphenols by the gut microbiota), and how the importance of potential active metabolites (such as urolithin) produced by the decomposition of polyphenols by gut microbiota is emerging. Based on the new perspective of gut microbiota, this review provides interesting insights into the mechanism of polyphenols in fatigue treatment and clarifies the potential of polyphenols as targets for anti-fatigue product development, aiming to provide a useful basis for further research and design.

## 1. Introduction

Fatigue has become a universal health problem in modern society. Persistent or severe fatigue not only affects people’s normal life, but also causes a variety of diseases related to biological regulation and immune system. However, the pathophysiology of fatigue is complex and not fully clear. Gut microbiota, or microorganisms in the gut, constitute a complex ecological community and have a great impact on the health of the host [1]. At present, the role of gut microbiota in disease and drug therapy has received more and more attention in the scientific community, and the bidirectional interaction between intestinal microbiota and host may be responsible for the progress of fatigue.

In recent years, research into the anti-fatigue effect of polyphenols has become an increasingly heated topic. A series of studies have shown that many polyphenols exhibit anti-fatigue effects, such as ellagitannins [2], tea polyphenols [3,4,5], rutin [6], curcumin [7,8,9], quercetin [10,11,12,13], soybean isoflavones [14], and so on. Polyphenols are abundant in foods such as fruits and vegetables, nuts, soybeans, tea, cocoa, and other foods, and are also found in many herbal medicines. Multiple preclinical and clinical studies have shown that they have antioxidant, anti-fatigue, anti-inflammatory, anti-diabetic, anti-cancer, neuroprotective, and anti-lipogenic properties [15,16,17,18,19]. However, the vast majority of polyphenols are difficult to be digested directly [20], but are broken down and metabolized by the gut microbiota in the human intestine into more active metabolites (such as urolithin, equol, etc.) [21,22]. Some bacterial species involved in polyphenol transformation have been found, and the regulatory effect of polyphenols on intestinal microbial ecology has been confirmed. Meanwhile, the potential biological activities of ingested polyphenols are affected by the ecology of individual gut microbiota [21]. Gut microbiota can metabolize polyphenols into more bioactive metabolites, which may in turn improve gut microbiota composition or regulate fatigue-related pathways to alleviate fatigue. However, at present, the activity of anti-fatigue polyphenols is mainly analyzed from the anti-oxidation and anti-inflammatory effects, and the changes in gut microbiota are rarely considered. Moreover, various evidence suggests that the interaction between gut flora, polyphenols, and host is a key point in the fatigue progress or treatment.

This review systematically summarizes the latest theoretical and clinical research results of the effect of gut microbiota on fatigue around the intestinal microecology. Here, we outline the relationship between gut microbiota and fatigue, including changes in the gut microbiota during fatigue and how they interact with the host. Next, we describe the interactions between the gut microbiota and polyphenols in fatigue therapy, which provides a useful basis for further research and design, and elucidates the potential of polyphenols to be developed as anti-fatigue products. Based on the new perspective of gut microbiota, we provide interesting insights into the mechanism of polyphenols in fatigue treatment.

## 2. Polyphenols

**Figure 1:**
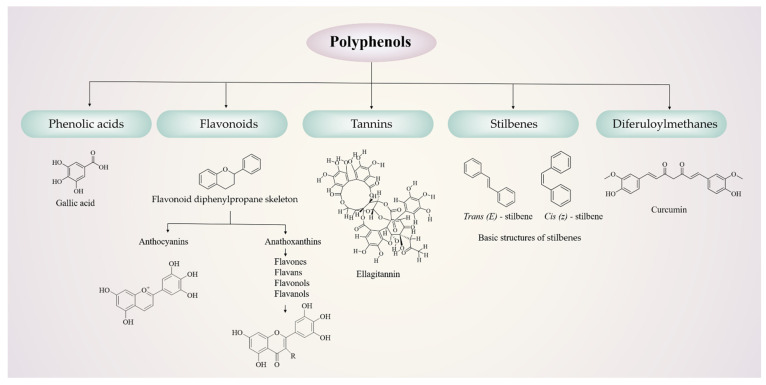
Classification of polyphenols.

Polyphenols are dietary antioxidants commonly found in plants and secondary metabolites of plants. They mainly exist in fruits, vegetables, nuts, soybeans, tea, cocoa, and wine. Chemically, polyphenols are characterized by aromatic rings with multiple hydroxyl groups. So far, 8000 identified compounds have been isolated in the scientific community [23]. Due to their complex structures, it is challenging to accurately classify and differentiate these compounds. According to the number of phenolic rings contained in polyphenolic compounds and the structural elements combined with these rings, they can be roughly divided into several categories: phenolic acids, flavonoids, tannins, stilbenes, and diferuloylmethanes [16,24]. The chemical structures are shown in Figure 1.

Although most polyphenols have a diverse and complex chemical structure, they can be decomposed by the intestinal microbiota into simpler compounds [25]. Polyphenols are usually conjugated with organic acids and sugars and generally not absorbed unless they are hydrolyzed. The gut microbiota can deconjugate glycosides, glucuronides, and organic acids to release corresponding aglycones [26]. The intestinal microbial conversion of polyphenols is divided into three major catabolic processes: hydrolysis (O-deglycosylations and ester hydrolysis), cleavage (ring and lactone fission; delactonization and demethylation), and reductions (dihydroxylation, double bond reduction and isomerization) [21,26]. Hydrolytic activity begins in the oral cavity and continues to enter the stomach through the digestive tract, where the size of food particles decreases, which promotes the release of phenolic compounds [27]. Studies have shown that only non-glycosylated phenolic compounds of polyphenols can be directly absorbed in the small intestine, accounting for about 5% to 10% of all polyphenols. A total of 90–95% of polyphenols are transferred to the colon, where they are decomposed and metabolized by intestinal microorganisms into more biologically active small molecules such as phenolic acids [28], derivatives of phenylacetic, phenylpropionic, phenylbutyric, and valeric acids and urolithins [29,30,31,32], and then further absorbed. Microbial conversions are different depending on the polyphenolic structure, polymerization degree, and spatial configuration. For example, flavonoids are metabolized by intestinal microorganisms and the C-ring is broken to produce hydroxy aromatic compounds mainly in the A-ring and B-ring phenolic acids. Flavonols are metabolized and the C ring is broken to produce 3,4- or 3,5-dihydroxyphenylacetic acid [33].

## 3. Interaction between Gut Microbiota and Host during the Progression of Fatigue

Gut microbiota is a microbial community living in the gastrointestinal tract [34]. There are about 100 trillion microorganisms, mainly composed of bacteria, but also a small number of viruses, protozoa, and eukaryotes, such as fungi. The intestinal microorganisms are mainly composed of six major bacterial phyla: actinobacteria (gram positive), Proteobacteria (gram negative), Verrucomicrobia (gram negative), and Fusobacteria (gram negative) [34,35]. In a healthy adult intestine, Bacteroidetes (gram negative) and Firmicutes (gram positive) account for more than 90% of the total. The gut contains a complex and dynamic microbial ecosystem, which is composed of beneficial bacteria, potentially harmful bacteria and other bacteria that may have two effects at the same time [36]. Potentially harmful bacteria include species of *Clostridium*, *Staphylococcus,* and *Veillonella* [27]. These species can produce potentially harmful products, such as toxins and carcinogens, which are associated with intestinal diseases, such as chronic inflammatory bowel disease and other immune-related diseases. Regarding beneficial bacteria, they mainly include *Lactobacillus* and *Bifidobacterium*, which can be used as antioxidants to regulate oxidative stress reaction in the metabolic process, reduce gas production, produce SCFAs, stimulate immunity, and have anti-tumor activity [37,38]. They play a key role in nutrition and disease prevention and are therefore often used as probiotics.

The human gut is a complex but stable micro-ecosystem, in which gut microbiota plays an important role in gut and human health. A delicate balance of bacteria is maintained in a healthy gut, and genetic and environmental specificities determine the type, quantity, and proportion of gut microbiota in a normal state. Under normal conditions, the gut microbiota and the human body are in a symbiotic relationship, which is of great significance for maintaining the immune function and metabolic balance of the human body [39]. However, various factors such as diet, age, drugs, environment, or living habits can break the balance of gut microbiota [40], and the imbalance of gut microbiota is an important incentive for the occurrence and development of various diseases in the human body. Therefore, a bidirectional interaction between gut microbiota and the host contributes to the progression of fatigue.

### 3.1. Fatigue and Imbalance of Gut Microbiota

In daily life, people often have physiological fatigue (central fatigue and exercise-induced fatigue), and pathological fatigue (chronic fatigue syndrome (CFS), disease-related fatigue) [18]. Studies have shown that when fatigue exists, in both rodents and humans, the increase in maleficent bacteria and the decrease in beneficial bacteria coexist. On the one hand, fatigue is associated with excessive lactate accumulation, energy deficit, and decreased central nervous system function, factors that are closely related to the metabolism of the gut microbiota. On the other hand, fatigue causes oxidative stress, inflammation, and dysfunction of the intestinal barrier, which are associated with dysregulation of the intestinal microbiota [41]. The gut microbiota associated with the occurrence of fatigue is shown in Table 1.

Here we discuss how 5-hydroxtryptamine (5-HT) plays a key role in central fatigue [42]. It is able to enter the brain and play a role as an inhibitory neurotransmitter, affecting the pituitary and mental state, leading to fatigue-related symptoms; 5-HT in the brain is closely related to the gut microbiota through the production of tryptophan, the precursor of 5-HT. The elevated level of tryptophan in blood makes it enter the central nervous system through the blood–brain barrier and finally be converted into 5-HT by tryptophan hydroxylase 2 (TPH2) [43]. Tryptophan is metabolized mainly through three different pathways, namely the kynurenine pathway, 5-HT pathway, and microbial metabolic pathway [44]. The gut microbiota plays an important role in tryptophan metabolism and can directly or indirectly regulate the 5-HT pathway in tryptophan metabolism. Moreover, some gut microbiota such as genera *Lactococcus*, *Lactobacillus*, *Streptococcus*, *Escherichia coli*, *Klebsiella*, and *Escherichia* are able to produce tryptophan synthetase to synthesize 5-HT in the intestine [45,46].

Gut microbiome is not only one of the mediating factors of exercise health effects, but also participates in the occurrence of exercise stress response and exercise fatigue [47,48]. It was found that there is an abundance of genera *Bifidobacterium* and *Megasphaera* in the gut microbiota during exercise-induced fatigue [49]. After 4 weeks of excessive swimming training in male rats, the diversity of gut microbiome at the phylum, family, and genus levels decreased, and the abundance changed significantly, among which the abundance of *Bacteroides* and *Helicobacter pylori* increased significantly [50]. Research shows that an important reason for exercise-induced fatigue is the excessive accumulation of L-lactic acid in skeletal muscle, which causes fatigue by reducing the pH value and energy supply of muscle [51]. In addition to glycolysis, the increase in L-lactic acid-producing bacteria in the gut microbiota, such as *Bifidobacterium breve Yakult*, *Escherichia coli*, and *Lactobacillus casei shirota* also leads to the increase in lactic acid content [52]. It has also been reported that the increase in proteobacteria is associated with oxidative stress and intestinal inflammation [53]. Fatigue can also cause intestinal injury, which is manifested in decreased intestinal barrier function, increased intestinal permeability, and decreased intestinal mucosal function. These results have been confirmed in the study of exercise-induced fatigue [54].

Many studies have shown that gastrointestinal factors are independent risk factors for CFS [55]. Rahel et al. [56] summarized that the main pathogenesis of CFS includes intestinal Dysbiosis, changes in intestinal brain axis activity, increased intestinal permeability accompanied by bacterial translocation, decreased levels of SCFAs, D-lactic acid acidosis, abnormal tryptophan metabolism, and low activity of the kynurenine pathway. Compared with healthy subjects, the gut microbiome of CFS patients changed, including the reduction in *Bifidobacterium* and *Escherichia coli*, and an increase in *Streptococcus faecalis* [57,58]. Giloteaux et al. [59] reported that the fecal bacterial culture of CFS patients was found to reduce the types of intestinal bacteria in CFS patients. Disruption of gut microbiota diversity was also associated with the severity of fatigue symptoms in CFS patients [60]. At the same time, CFS patients have increased sensitivity to intestinal microecological changes [61]. One of the causes of CFS is that the increased intestinal permeability promotes the absorption of D-lactate acid from the intestine to the body [43,62]. In clinical cases, almost all CFS patients have increased intestinal permeability [63].

The relationship between fatigue and gut microbiome is not only a correlation or potential causal relationship, but also a covariant relationship in the process of disease. Changes in the gut microbiome can affect the progress of the disease, and physiological changes caused by fatigue can also change the intestinal tract, the type and abundance of the microbiota, and make the patient more susceptible to changes in the microbiota.

**Table 1:** The gut microbiota involved in fatigue-induced damage [41].

Gut Microbiota *	Factor Related to Fatigue	Target	Effect	Reference(s)
*Escherichia* *Streptococcus* *Enterococcus*	5-HT	Brain	Central fatigue	[43,62]
*Bifidobacterium breve Yakult* *Lactobacillus casei Shirota* *Escherichia coli*	L-lactic acid	Blood	Imbalance of muscle and blood pHs; reduction in muscle function and muscle contractility; exercise-induced fatigue	[52]
*Lactobacillus acidophilus* *Lactobacillus fermentum* *Lactobacillus delbrueckii* *subsp. Lactis* *Lactobacillus buchneri* *Streptococcus bovis* *Enterococcus*	D-lactic acid	Blood	Metabolic disorders, direct or indirect neurotoxic effects; CFS	[64]

* The names of bacteria at the genus or species level are in italics, and those at the phylum or family level are in the normal style.

### 3.2. Gut Microbiota Shows Beneficial Effects against Fatigue

The gut microbiome itself and its metabolites play an important role in the host’s physiology, metabolism, nutrition, and immunity [65,66,67,68], and have shown positive effects in anti-fatigue. For example, Hsu et al. found that normal rats, rats containing only Bacteroides fragilis, and germ-free rats shortened the exhaustive swimming time in turn, and the exhaustive time was positively correlated with the abundance of gut microbiome in rats [66]. Valenzano et al. found that middle-aged killifish transplanted with fecal bacteria from young killifish have similar gut microbiota structures, improved exercise capacity, and activity levels similar to those of young killifish [69]. The anti-fatigue mechanism of gut microbiome may be related to factors such as the gut microbiome itself and its metabolites can improve the host’s energy metabolism process, improve the intestinal mucosal barrier function, and enhance the host’s immune function.

The main beneficial intestinal probiotics in the human body are: *Bifidobacterium*, *Lactobacillus*, *Prevotella*, *Bacteroides*, *Akkermansia*, and *Lactobacillus* which are important probiotics in the intestinal tract of organisms [70,71]. They can not only decompose and metabolize prebiotics in the intestinal tract and produce metabolites that are beneficial to the intestinal microecology, but also inhibit the reproduction of harmful bacteria to a certain extent, to improve the intestinal environment, so it can maintain immune balance and enhance intestinal barrier function. Lactobacillus, a genus that predominates in the intestinal tract of endurance athletes [72], promotes protein utilization and increases the content of branched-chain amino acids (BCAAs) that maintain muscle energy homeostasis, thus delaying the onset or development of fatigue [73]. *Prevotella*, *Bacteroides*, *Treponema,* and *Butyrivibrio* and other degrading-fibers bacteria are known to produce high levels of SCFAs [74,75]. SCFAs can provide energy for intestinal epithelial cells, and have physiological functions such as energy regulation, maintenance of fat homeostasis and intestinal barrier integrity, and regulation of the blood–brain barrier (BBB) [76,77,78,79], thereby playing a beneficial role in anti-fatigue. In a previous study, *Veillonella atypica* isolated from the fecal samples of marathon runners was inoculated into mice, which significantly increased the time for mice to run exhausted on the treadmill. *V. atypica* can improve sports performance by converting exercise-induced lactic acid into propionic acid through metabolism [80]. In addition, some specific flora in the gut microbiome can convert the exercise metabolite-lactic acid into propionic acid, and participate in the exercise again to provide energy to achieve the effect of delaying fatigue [80]. *Bacillus* and *Saccharomyces* produce noradrenalin, and *Bacillus* also produces dopamine [45]. Norepinephrine and dopamine are both excitatory neurotransmitters, which can promote exercise performance and thus reduce fatigue symptoms caused by exercise. Probiotics such as *Bifidobacterium* and *Lactobacillus* can enhance the intestinal mucosal barrier function, stimulate the proliferation of epithelial cells, stimulate the production of intestinal sIgA, and maintain the intestinal microecological balance [81]. *Akkermansia muciniphila* is a common human intestinal mucus-degrading bacterium with promising probiotic activity [82]. *A. muciniphila* can produce SCFAs by decomposing mucins, stimulate goblet cells to produce more mucus, and thus supplement or maintain the integrity of intestinal barrier. In addition, *A. muciniphila* may reduce the abundance of Firmicutes and *Clostridia*, thus promoting intestinal homeostasis [83]. Enhanced gut barrier function prevents lactate from entering the bloodstream, thereby inhibiting excessive accumulation of lactate in muscles.

## 4. Gut Microbiota–Polyphenols Interaction during Treatment of Fatigue

**Figure 2:**
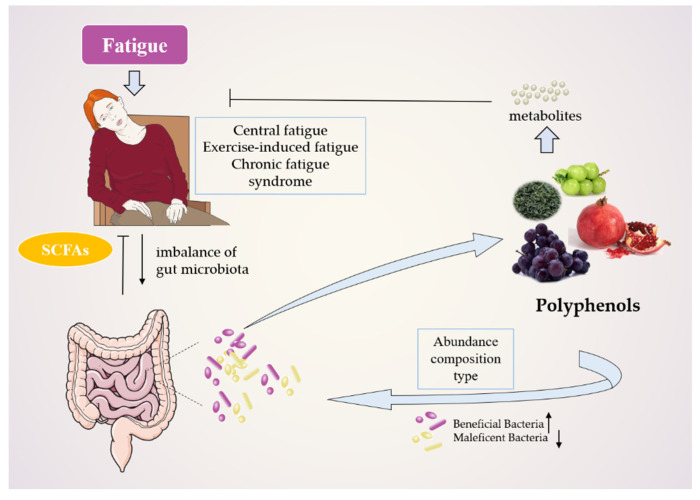
Occurrence and treatment of fatigue: interaction of polyphenols, host, and gut microbiome.

In fatigue treatment, the gut microbiota, host, and polyphenols interact with each other. Polyphenols regulate the composition of beneficial and harmful bacteria in the gut (as shown in Figure 2). The gut microbiota decomposes polyphenols to produce highly antioxidant or anti-inflammatory metabolites, produce SCFAs, maintain the integrity of intestinal barrier, inhibit intestinal inflammation, and stimulate the production of neurotransmitters regulating the central nervous system. Therefore, the mechanism of anti-fatigue polyphenols is mainly related to antioxidant and anti-inflammatory, protecting intestinal integrity, regulating energy metabolism, and producing anti-fatigue metabolites.

### 4.1. Polyphenols Regulate the Composition of Beneficial and Maleficent Bacteria

The progression of fatigue is often accompanied by an imbalance in gut microbiota, and anti-fatigue polyphenols can improve the composition of gut microbiota. Moreover, increasing the abundance of beneficial bacteria helps supply energy and maintain the integrity of the intestinal barrier. A number of in vitro and in vivo studies have shown that polyphenols can inhibit the growth of *Clostridium* spp., (*C. histolyticum*), *Pseudomonas* spp., *Salmonella* spp., *Bacillus* spp., *Escherichia coli*, *Helicobacter pylori*, and increase some beneficial bacterial groups, such as *Lactobacillus* spp., *Bifidobacterium* spp., *Akkermansia* spp. (*A. muciniphila*) and *Faecalobacterium* spp. (*F. prausnitzii*), some of which can metabolize polyphenols [23,24,84,85,86]. Some polyphenols with high anti-fatigue potential are described below (as shown in Table 2).

#### 4.1.1. Effects of Polyphenol Mixtures on Gut Microbiota

##### Ellagitannins

Ellagitannins are tannins formed by hexahydroxybiphenyl diacid and its derivatives as well as polyols through glycosidic or ester bonds, which produce ellagic acid after hydrolysis. In the mouse experiment, oral administration of jabuticaba seed extract rich in ellagic acid and ellagitannins can increase the ratio of Bacteroidetes to Firmicutes, promote the growth of gut microbiota, and be beneficial to human health [87]. In addition, ellagine tannin and concentrated tannin can promote the growth of *lactobacilli* and *bifidobacterial* [88]. In the case of ellagitannins, this prebiotic effect has been confirmed in human flora in vitro [89], animal models in vivo, and recently in humans [90]. Interestingly, pomegranate ellagine increased the amount of *A. muciniphila* in the feces of obese mice in a manner similar to that of cranberry extract rich in concentrated tannins (proanthocyanidins) [90,91]. Therefore, ellagitannin relieves fatigue by producing prebiotic-like effects, increasing the proportion of beneficial bacteria in the intestinal tract, improving the imbalance of gut microbiota, supplying energy to the body, and protecting the intestinal barrier.

##### Anthocyanins

Anthocyanins are flavonoids formed by the combination of anthocyanin and sugar by glycosidic bonds, it is a kind of natural pigment. Hidalgo et al. [92] found that the anthocyanin mixture can significantly promote the growth of *Bifidobacterium*, *Lactobacillus,* and *Enterococci* in vitro fermentation experiments. High-purity blueberry anthocyanins (96.8% purity) can affect the microbial diversity of human gut microbiome. After anaerobic fermentation in vitro for 12 h, it can increase the levels of *Bifidobacterium*, *Ruminococcus*, *Clostridium* IV, and Proteobacteria, and this effect is especially obvious for probiotics such as bifidobacteria [93]. These studies showed that rational use of anthocyanins can improve the activity of intestinal probiotics. After 12 weeks of continuous feeding with 200 mg/kg *Lycium barbarum* L. anthocyanins per day, the liver antioxidant enzyme system was activated, the intestinal barrier zonula occludens 1 (Zo-1), occludin, tight junction protein, and mucin. The mRNA expression of protein-1 was significantly increased, and gut microbiota such as *Barnesiella* and *Alistipes* were all regulated [94] Taken together, anthocyanins may alleviate fatigue by exerting multiple bioactivities such as anti-oxidative stress, anti-inflammatory, intestinal barrier, and gut microbiome.

##### Tea Polyphenols

Tea polyphenols are the general name of polyphenols in tea, mainly including catechins, 4-hydroxyflavanols, anthocyanins, flavonoids, flavonols, and phenolic acids, among which catechins and flavonoids are the main substances [95]. As early as 1993, it was reported that tea polyphenols can promote the growth of *Bifidobacterium* and *Lactobacillus* in chicken feces, and inhibit *Escherichia coli* [96]. Studies have found that black tea polyphenols (rich in theaflavins, thearubigins, and their mono- and di-gallates) change the composition of gut microbiota, inhibit Firmicutes, and promote Proteobacteria, that is, increase the levels of *Klebsiella*, *Enterococci*, and *A. muciniphila*, and reduce the levels of *Victivallis*, *B.coccoides,* and *Anaeroglobus* [97]. Catechins in Oolong tea and green tea may have prebiotic-like activities and can be used as functional food ingredients to prevent gut microbiota imbalance [98,99,100]. Moreover, the concentration of SCFAs in the culture supplemented with catechin was relatively higher than that in the control [101]. Research on polyphenols in black tea and green tea show that they are able to inhibit the growth of a variety of pathogens in vitro, including *Helicobacter pylori*, *Staphylococcus aureus*, *Escherichia coli* O157: H7, *Salmonella typhimurium* DT104, *Pseudomonas aeruginosa* and so on [102]. Liao et al. [103] found that tea polyphenols can significantly increase the abundance of *bifidobacteria* in mice. A similar result was also observed in a clinical study. Ten volunteers had increased *bifidobacteria* in their feces after drinking green tea for 10 days [104]. The effect of tea catechins on bacterial growth and metabolism depends on the structure, dose, and microbial strain of polyphenols, which can interact with the bacterial cell surface, inhibit enzyme activity, and thus affect energy metabolism [29]. In general, tea catechins, as a supplement of prebiotics, can regulate the composition of gut microbiota by enriching beneficial bacteria and inhibiting some pathogenic bacteria. As a result, the application of tea catechins may be beneficial to prevent and alleviate fatigue.

##### Grape Polyphenols

Grape polyphenols exist in grape skins and grape seeds, mainly including phenolic acids, flavanols, flavanones, flavonols, anthocyanins, and resveratrol. Studies have shown that polyphenols in red wine and grape significantly increase the abundance of *A. muciniphila*. In addition, polyphenols in red wine during in vitro fermentation increased the relative abundance of *Klebsiella*, *Victivallis*, *Cloacibacillus*, and *Alistipes*, and decreased the abundance of *Bacteroides*, *Blautia cocoids*, *Anaeroglobus*, and *Subdoligranulum* [97]. It is also reported that in vitro fermentation of grape and grape seed polyphenols can inhibit the growth of *Clostridium perfringens* and *Clostridium histolyticum* [105,106]. Oral red wine polyphenols can increase the abundance of *Bacteroides*, *Lactobacillus* spp., and *Bifidobacterium* in the intestinal microbiome, and reduce the abundance of *Clostridium* spp. [107]. In an animal experiment by Ying et al. [108], six female pigs were fed grape seed extract for 6 days. The results showed that dietary supplementation of grape seed extract can alter gut microbiota, with significant increases in Lachnospiraceae, Clostridiales, *Lactobacillus* and Ruminococcaceae. Queipo-Ortuno [109] found that daily consumption of red wine polyphenols for four weeks significantly increased the relative abundance of genera *Bifidobacterium* and *Lactobacillus* genera, promoting healthy development of human gut microbes. In addition, red wine polyphenols are also beneficial to the growth of *Enterococci*, *Prevotella*, *Bacteroides*, and *Eggerthella lenta*, but alcohol has no such effect. Among the many gut microbiomes, the most sensitive to polyphenols is *Bifidobacterium*.

##### Other Polyphenol Mixtures

Tzounis et al. [110] found that the alcohol extract of cocoa flavone can increase the abundance of *Bifidobacterium* and *Lactobacillus* in human intestine. Massot Cladera et al. [111] found that the cocoa polyphenol extract can significantly reduce the relative abundance of *Bacteroides*, *Clostridium,* and *Staphylococcus*. Bialonska et al. [89] published articles that pomegranate polyphenol extract can promote the growth of *Bifidobacterium*, *Lactobacillus,* and some bacteria producing SCFAs in human intestinal tract. In an in vitro study, olive pomace powder prevented the growth of pathogenic bacteria such as species *Bacillus cereus* and *Listeria monocytogenes* [112]. In the process of microbial fermentation in mice and human intestines, the antioxidant activity of polyphenol enriched in bayberry and mulberry increased [113,114]. A study used an in vitro intestinal model to evaluate the potential prebiotic activity of seaweed polyphenols, which significantly increased the abundance of Firmicutes and facilitated the production of SCFAs [115].

#### 4.1.2. Effects of Polyphenolic Monomer Compounds on Gut Microbiota

##### Curcumin

Curcumin is a kind of polyphenol isolated from the rhizomes of Curcumaceae and Araceae plants. There is evidence that curcumin can restore the intestinal barrier function by regulating the cell bypass permeability of the intestinal barrier system [116], and the serum lactic acid accumulated during exercise can enter the intestinal lumen through the epithelial barrier, thereby affecting athletic performance [80]. Curcumin can enhance skeletal muscle performance by increasing cAMP levels and regulating mitochondrial biogenesis [117]. The poor solubility of curcumin, poor intestinal absorption, and fast metabolism and systemic elimination limit the use of curcumin. One possible mechanism by which curcumin exerts its biological activity is to regulate the intestinal microbiota as a prebiotic. There is evidence that high concentration of curcumin can reduce the dysbiosis of microbial flora. Therefore, the high content of curcumin is the key to the decrease in unfavorable bacteria or the increase in probiotics [118]. Chen et al. [119] found that the curcumin extract of nano bubbles can increase the proportion of Proteobacteria and lactobacteria in the cecum of mice, but it does not reduce the microbial diversity, indicating that it changes the composition of intestinal microorganisms and increases the abundance of lactobacilli. By fermenting lactic acid into butyrate or other SCFAs, it can improve sports performance and reduce physical fatigue. Lactic acid produced during continuous exercise enters the microbiota and be converted into SCFAs, which are known to improve athletic performance [120,121]. The effects of curcumin (as well as resveratrol and simvastatin) have also been studied in animals affected by Toxoplasma gondii. The results showed that the number of proinflammatory *Enterobacteria* and *Enterococci* decreased in curcumin-, resveratrol-, and simvastatin-treated animals, and the number of *Lactobacilli* and *Bifidobacteria* with anti-inflammatory effects slightly increased [122].

##### Quercetin

Quercetin belongs to the flavonol group. In an in vitro study, Xue et al. [123] found that quercetin was added to liquid medium and co-fermented with human fecal flora for 24 h. The results showed that quercetin can significantly inhibit the growth of Bacteroidetes and Firmicutes. In addition, it was also found that quercetin can affect the function of the bacteria on polysaccharide metabolism and energy metabolism by regulating the composition of gut microbiome. It is well-known that insufficient energy supply is a major cause of fatigue. In addition to the above reports, Firrman et al. [124] found that quercetin can increase the relative abundance of *Bifidobacterium* and *Lactobacillus*, and inhibit the growth of *Escherichia coli*, *Clostridium histolyticum*, and *Enterococci*. Etxeberria et al. [125] fed rats quercetin alone to significantly modulate the composition of the rat gut microbiome.

##### Resveratrol

Resveratrol belongs to stilbene compounds, which may play an anti-fatigue role by increasing the abundance of beneficial bacteria and inhibiting the growth of harmful bacteria. Larrosa et al. [126] found that resveratrol can increase the relative abundance of *Bifidobacterium* and *Lactobacillus* in rat intestine, and protect colon mucosa. Similarly, Qiao et al. [127] found that resveratrol can significantly increase the ratio of Bacteroidetes to Firmicutes in mice. At the same time, it significantly inhibited the growth of *Enterococci* and increased the relative abundance of Bifidobacterium and Lactobacillus. It was found that resveratrol supplementation can increase the ratio of Bacteroidetes to Firmicutes in the cecal microbiota, thereby improving fatigue and exercise intolerance during heart failure in mice [128].

##### Other Polyphenolic Monomer Compounds

Lee et al. studied the effect of the main components of tea polyphenols: epicatechin, catechin, caffeic acid, and gallic acid on intestinal microorganisms through in vitro fermentation. These flavanols inhibit the growth of pathogenic bacteria such as *Clostridium perfrigens*, *Clostridium difficile,* and *Bacteroides*, while the relative abundance of probiotics such as *Bifidobacterium* and *Lactobacillus* increases [129]. Gallic acid can induce changes in the composition and activity of gut microbiota to be more favorable, such as increasing the abundance of probiotics such as Lactobacillaceae and Prevotellaceae families, including the production of SCFAs in the colon [130], and inhibiting the growth of pathogenic bacteria such as phyla Firmicutes and Proteobacteria. Tzounis also reported [131] that epicatechin and catechol can increase the relative abundance of *Bifidobacterium* and *Lactobacillus*, and inhibit the growth of *Escherichia coli*, *Clostridium histolyticum,* and *Enterococci*.

**Table 2:** Effects of different polyphenols on gut microbiota.

Chemicals	Polyphenol Source	Regulation of Gut Microbiota *	Reference(s)
Ellagic acid, ellagitannins	Jabuticaba seeds	Firmicutes ↑	[87]
		Bacteroidetes ↑	
		Proteobacteria ↑	
Ellagitannins	Pomegranate	*A. muciniphila* ↑	[88]
Anthocyanins		* Bifidobacterium * ↑	[92]
		* Lactobacillus * ↑	
		* Enterococci * ↑	
Catechins	Tea	Interacting with the surface of bacterial cells and inhibits enzyme activity, thus affecting energy metabolism	[29]
Tea polyphenols	Green tea	* Bifidobacterium * ↑	[96]
		* Lactobacillus * ↑	
		* Escherichia coli * ↓	
	Black tea	* A. muciniphila * ↑	[97]
		* Klebsiella * ↑	
		* Enterococci * ↑	
		* Victivallis * ↓	
		* B. coccoides * ↓	
		* Anaeroglobus * ↓	
Grape polyphenols	Grape	* A. muciniphila * ↑	[97]
		* Klebsiella * ↑	
		* Victivallis * ↑	
		* Cloacibacillus * ↑	
		* Alistipes * ↑	
		* Bacteroides * ↓	
		* Blautia coccoides * ↓	
		* Anaeroglobus * ↓	
		* Subdoligranulum * ↓	
	Grapes, grape seeds	* Clostridium perfringens * ↓	[105,106]
		* Clostridium histolyticum * ↓	
Polyphenols	Red wine	* Bacteroides * ↑	[107]
		* Lactobacillus spp. * ↑	
		* Bifidobacterium * ↑	
		* Clostridium spp. * ↓	
Cocoa flavone	Cocoa	* Bifidobacterium * ↑	[110]
		* Lactobacillus * ↑	
Cocoa polyphenols	Cocoa	* Bacteroides * ↓	[111]
		* Clostridium * ↓	
		* Staphylococcus * ↓	
Polyphenols and capsinoids	Sweet pepper	Bacteriodetes ↑	[112]
		Firmicutes ↓	
Polyphenols and flavonoids	Dendropanax morbifera leaf	* Bacteroides * ↑	[132]
		* Allobaculum * ↑	
Polyphenols	Plinia jaboticaba berry	* Lactobacillus * ↑	[133]
		* Bifidobacterium * ↑	
		Enterobacteriaceae ↑	
Pomegranate polyphenol	Pomegranate	* Bifidobacterium * ↑	[89]
		* Lactobacillus * ↑	
Gallic acid		Lactobacillaceae ↑	[130]
		Prevotellaceae families ↑	
		SCFAs ↑	
Curcumin, resveratrol		Enterobacteria ↓	[122]
		Enterococci ↓	
		Lactobacilli ↑	
		Bifidobacteria ↑	
Curcumin	Rhizomes of curcumaceae and Araceae	Regulating cell, bypass permeability of intestinal barrier system the Bacteroidetes to Firmicutes ratio ↓	[116]
Quercetin		*Bifidobacterium* ↑	[123,124]
		*Lactobacillus* ↑	
		*Escherichia coli* ↓	
		*Clostridium histolyticum* ↓	
		*Enterococci* ↓	
Resveratrol		The Bacteroidetes to Firmicutes ratio ↑	[126,128]
		* Bifidobacterium * ↑	
		* Lactobacillus * ↑	

*, ↑, indicates an increase in bacterial abundance; ↓, indicates the abundance of bacteria is reduced.

### 4.2. Gut Microbiota-Mediated Metabolism Modulates the Biotransformation of Polyphenols

Due to poor absorption capacity, high metabolic rate and fast elimination speed, polyphenols may not be the most bioactive compounds in the human body. Studies have shown that non-digestible polyphenols can improve the composition of gut microbiota, which is related to metabolism into more bioactive secondary metabolites [134]. By producing glycosidases and other enzymes to catalyze phase I reactions (such as oxidation or hydrolysis), gut microbiota converts phytochemicals into small molecules that are easily absorbed or metabolites with pharmacological effects [26,135], such as urolithin. For polyphenols, the metabolism of each specific compound by gut microbiota depends not only on its general chemical structure, but also on the number, type and position, stereoisomerism, and polymerization degree of specific functional groups. In addition, specific bacterial species/strains are required to carry out specific transport of internal molecules, as well as specific enzymatic mechanisms to catalyze different reactions on the polyphenol core.

The metabolism of polyphenols in the body does not play a role in most cases by a single flora, but a variety of gut microbes interact and work together to complete complex metabolic processes. Quercetin can be converted into 3,4-dihydroxyphenylacetic acid, 3,4-dihydroxyphenylacetic acid under the action of gut microbiome such as Streptococcus S-2, Lactobacillus L-2, Bifidobacterium B-9 and Bacteroides JY-6; 4-Dihydroxybenzoic acid, 4-hydroxybenzoic acid, 3-(3-hydroxybenzene) propionic acid and other small molecules are absorbed and utilized by the body [136]. Rutin is hydrolyzed by α-rhamnosidase and β-glucosidase in the intestinal tract, and then rutose is removed to form quercetin, which is then reduced and hydrogenated to form dihydroquercetin, and then under the action of isomerase to form 3, 4-dihydroxyphenyl-valerolactone, and finally oxidatively cracked to form 3,4-dihydroxyphenylacetic acid and phloroglucinol [137]. The daidzein is hydrolyzed by β-glucosidase to form daidzein, which is then reduced to R(−)-dihydrodaidzein by daidzein reductase, and then converted to S(−)-dihydrodaidzein by the action of racemase. Hydrogen daidzein is further converted into S(−)-tetrahydrodaidzein under the action of dihydrodaidzein reductase, which is finally converted into S(−)-equol by deketonization reaction [138]. In conclusion, gut microbiome can promote the release of more active polyphenol metabolites in the human body, enhance the biological activity of polyphenols and their metabolites, and reduce the toxicity and production of harmful substances. It plays an important role in metabolism, absorption, bioavailability, and efficacy in the body. Taking urolithin as an example, this paper explores how gut microbiota regulates the biotransformation of polyphenols to exert anti-fatigue effects.

#### Urolithin

**Figure 3:**
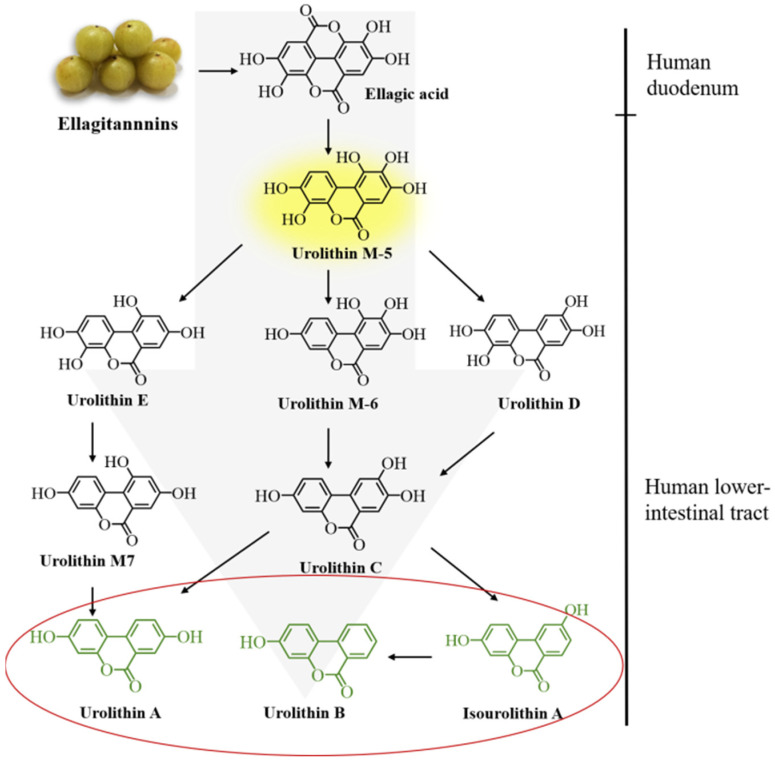
The catabolic pathway of ellagitannin to urolithin [143].

Ellagic acid and ellagitannins have a variety of biological activities such as anticancer, anti-diabetes, and prevention of cardiovascular and neurodegenerative diseases, but their intestinal absorption and bioavailability are very low, leading to being either directly eliminated in feces or converted into a more bioavailability derivative urolithin [22,139]. Ellagitannins are hydrolyzed into ellagic acid by tannase in the intestine. Under the action of gut microbiota, ellagic acid is further converted into pentahydroxyurolithin (urolithin M5) through the cleavage and decarboxylation of lactone ring, which is a key intermediate for the production of different urolithins. Starting from urolithin M5, continuous dehydroxylation eventually transforms ellagic acid into major metabolites that can be detected in vivo: dihydroxyurolithin (urolithin A), isourolithin A and 3-hydroxyurolithin (urolithin B) (as shown in Figure 3). Urolithin A and Urolithin B are the most abundant end products. Recently, two strains of bacteria capable of producing urolithin intermediates (urolithin M5, urolithin M6 and urolithin C) were isolated from human fecal samples. These two strains belong to the Eggerthellaceae family and are named as *Gordonibacter urolithinfociens* (DSM 27213^T^) and *G Pamelaeae* (DSM 19378^T^) [140,141]. A bacterium capable of metabolizing isourolithin a (the final product), also from the Eggerthellaceae family, was named *ellagibacter isourolithinifaciens* (DSM 104140^T^) [142]. However, bacteria that can metabolize urolithin A and urolithin B have not been isolated yet.

**Figure 4:**
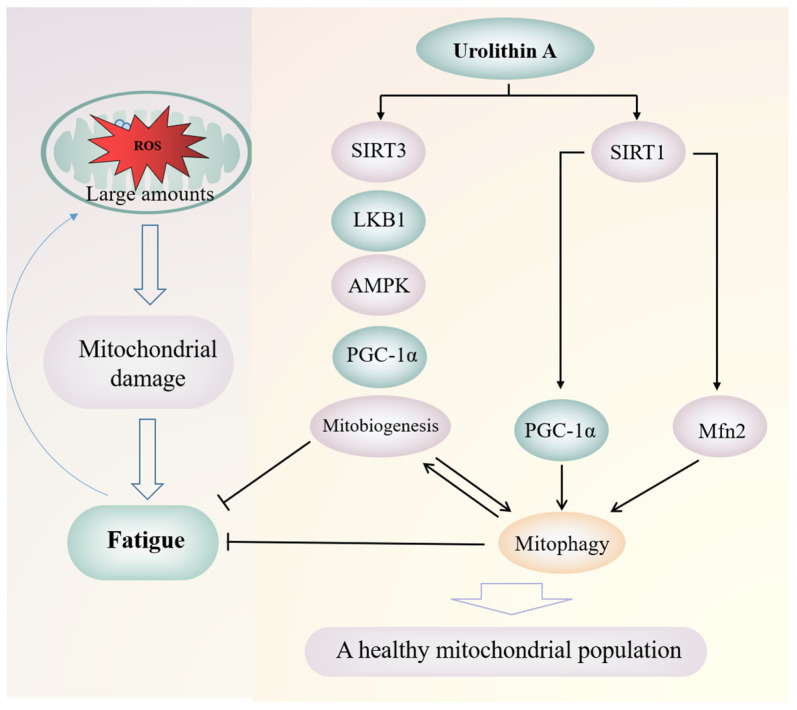
The maintenance effect of urolithin A on mitochondrial homeostasis under fatigue.

The bioavailability of urolithin is much higher than that of its precursor compound, so the biological activities of ellagic acid and ellagitannins may be mediated by urolithin [144]. In fact, there is evidence that urolithin has higher anti-inflammatory, antioxidant, and antiproliferative abilities than its precursors, which also supports this hypothesis [145]. Urolithin A has been proved to improve mitochondrial activity and muscle function (as shown in Figure 4), which may be due to its induction of mitochondrial autophagy and antioxidation [143,146,147]. The mammalian C2C12 myoblasts and Mode-K intestinal cells treated with urolithin A showed a dose-dependent increase in autophagy and mitochondrial autophagy biomarkers [146]. A rodent study also showed that urolithin A supplementation increased average running endurance by 42% [146]. Zhao et al. [148] reported that urolithin A enhanced the SIRT3 promoter activity of Caco-2 cells; urolithin A can increase ATP and NAD+levels, cause activation of SIRT1 promoter, and affect SIRT1-PGC-1 α access [149]. SIRT1 regulates the expression of mitofusin2 (Mfn2) and subsequent mitochondrial autophagy [150]. Some studies also showed that urolithin A increased the expression of Mfn2 in the pathway of inducing mitochondrial autophagy [151]. Mitochondrial health is closely related to fatigue [18]. Activation of SIRT, AMPK, and PGC1-α and inhibition of mTOR tend to induce mitophagy and mitochondrial biogenesis to maintain mitochondrial health [152]. Urolithin also has an effect on gut microbiota. A study on rat intestinal inflammation model shows that oral urolithins can promote the growth of Lactobacillus and Bifidobacterium [90].

Studies have shown that not all individuals can convert ellagic acid and ellagitannins into urolithin [153]. Individuals that only produce urolithin A and its conjugates are classified as type A metabolism, individuals that only produce urolithin B or isourolithin A and individuals that cannot produce any form of urolithin are classified as type B metabolism and type 0 metabolism, respectively [154]. Some clinical studies have shown that compared with other urolithin metabolic types, type A metabolists have a body mass index (BMI) within the normal range (1925 kg/m^2^), better intestinal health, and lower baseline risk values of serum CVD biomarkers [155,156]. Therefore, type A metabolism may be a favorable metabolic type among urolithin metabolic types. As mentioned above, urolithin A is an important active substance that plays an anti-fatigue role. Therefore, promoting the growth and formation of key bacteria of type A metabolism is conducive to alleviating fatigue. However, the proportion of type A metabolists decreases with age [154], from 70% at first to only 40% of the elderly [31]. Improving the composition of gut microbiota may be an important way to increase the transformation of ellagic acid to urolithin A. Studies have shown that long-term or high-dose use of ellagic acid or ellagitannins can transform some people with type 0 metabolism into type A metabolism or type B metabolism [157].

## 5. Discussion

Gut microbiota is a new research field, which provides a new way for people to understand many diseases. It plays an important role in the cause and effect of fatigue and the mechanism of anti-fatigue. Fatigue can lead to an imbalance of gut microbiota. Moreover, the destruction of gut microbiota and related metabolites can promote the development of fatigue, thereby aggravating fatigue-related injuries. However, further research is needed to determine the causal relationship between gut microbiota and fatigue. The three common types of fatigue, exercise-induced fatigue, mental fatigue, and CFS, all have interactions between gut microbiota and host. According to the available data, excessive lactate accumulation, energy deficit, and decreased central nervous system function are related to the metabolism of gut microbiota. Among the harmful effects of fatigue, oxidative stress, inflammation, and dysfunction of the intestinal barrier, which are associated with imbalance of the intestinal microbiota. However, only the relationship between chronic fatigue syndrome and gut microbiota has been studied in depth, while the other two types of fatigue have been studied less and shallowly, limited to several markers such as 5-HT and lactic acid, and lack of further demonstration.

Our previous research also mentioned the anti-fatigue effect and mechanism of polyphenols [18], and polyphenols are mostly derived from food and herbs, which are very safe and easy to obtain. Therefore, some polyphenols, such as tea polyphenols, tannins, urolithin, etc., are reasonable as potential targets for the development of anti-fatigue products, and more anti-fatigue polyphenols can be further developed. However, changes in gut microbiota have not been taken into account when studying most anti-fatigue polyphenols, and it should be considered as an important factor. In the few studies available, *Bifidobacterium* is the most vulnerable among many intestinal microorganisms, and almost all relevant reports note that polyphenols can significantly increase its abundance. In addition, many polyphenols can also significantly promote the growth of *Lactobacillus*, and *Akkermansia*. In addition to the three probiotics that have been significantly promoted, a high proportion of literatures reported that polyphenols can inhibit the growth of pathogenic bacteria such as *Clostridium perfringens*, *Clostridium histolyticum*, *Clostridium difficile,* and *Escherichia coli*. Moreover, both polyphenol mixtures and polyphenol monomers have a great impact on the composition of gut microbiota. Polyphenols can relieve fatigue by increasing the relative abundance, species, and activity of beneficial intestinal bacteria or inhibiting the abundance or activity of harmful bacteria. So as to produce beneficial SCAFs, protect the intestinal barrier, strengthen the immune system, and alleviate the damage caused by fatigue. However, the research results of gut microbiota change are mostly at the level of phylum or genus, and few at the level of species, which is not conducive to the targeted development of more effective products to regulate gut microbiota. The identification of anti-fatigue-related intestinal bacteria at the species level needs further research. The research on the metabolism of anti-fatigue polyphenols is mostly limited to the regulation of polyphenols as prebiotics on the composition of the overall gut microbiota, while the role of polyphenols as more bioactive metabolites generated by gut microbiota metabolism has been ignored. The interaction among them is interesting and meaningful, but only urolithin has some clues at present.

## Data Availability

Not applicable.

## References

[1] Lozupone C.A., Stombaugh J.I., Gordon J.I., Jansson J.K., Knight R. (2012). Diversity, stability and resilience of the human gut microbiota. Nature.

[2] Belcaro G., Saggino A., Cornelli U., Luzzi R., Dugall M., Hosoi M., Feragalli B., Cesarone M.R. (2018). Improvement in mood, oxidative stress, fatigue, and insomnia following supplementary management with Robuvit®. J. Neurosurg. Sci..

[3] Liu L., Wu X., Zhang B., Yang W., Li D., Dong Y., Yin Y., Chen Q. (2017). Protective effects of tea polyphenols on exhaustive exercise-induced fatigue, inflammation and tissue damage. Food Nutr. Res..

[4] Teng Y.S., Wu D. (2017). Anti-Fatigue Effect of Green Tea Polyphenols (−)-Epigallocatechin-3-Gallate (EGCG). Pharmacogn. Mag..

[5] Singal A., Kaur S., Tirkey N., Chopra K. (2005). Green tea extract and catechin ameliorate chronic fatigue-induced oxidative stress in mice. J. Med. Food.

[6] Su K.Y., Yu C.Y., Chen Y.W., Huang Y.T., Chen C.T., Wu H.F., Chen Y.L. (2014). Rutin, a flavonoid and principal component of saussurea involucrata, attenuates physical fatigue in a forced swimming mouse model. Int. J. Med. Sci..

[7] Huang W.C., Chiu W.C., Chuang H.L., Tang D.W., Lee Z.M., Wei L., Chen F.A., Huang C.C. (2015). Effect of curcumin supplementation on physiological fatigue and physical performance in mice. Nutrients.

[8] Chen Y., Wang J., Jing Z., Ordovas J.M., Wang J., Shen L. (2022). Anti-fatigue and anti-oxidant effects of curcumin supplementation in exhaustive swimming mice via Nrf2/Keap1 signal pathway. Curr. Res. Food Sci..

[9] Agarwal K.A., Tripathi C.D., Agarwal B.B., Saluja S. (2011). Efficacy of turmeric (curcumin) in pain and postoperative fatigue after laparoscopic cholecystectomy: A double-blind, randomized placebo-controlled study. Surg. Endosc..

[10] Chen X., Liang D., Huang Z., Jia G., Zhao H., Liu G. (2021). Anti-fatigue effect of quercetin on enhancing muscle function and antioxidant capacity. J. Food Biochem..

[11] Mahoney S.E., Davis J.M., Murphy E.A., McClellan J.L., Pena M.M. (2014). Dietary quercetin reduces chemotherapy-induced fatigue in mice. Integr. Cancer Ther..

[12] Bigelman K.A., Chapman D.P., Freese E.C., Trilk J.L., Cureton K.J. (2011). Effects of 6 weeks of quercetin supplementation on energy, fatigue, and sleep in ROTC cadets. Mil. Med..

[13] Wu J., Gao W., Wei J., Yang J., Pu L., Guo C. (2012). Quercetin alters energy metabolism in swimming mice. Appl. Physiol. Nutr. Metab..

[14] Liu Y., Zhou Y., Nirasawa S., Tatsumi E., Cheng Y., Li L. (2014). *In vivo* anti-fatigue activity of sufu with fortification of isoflavones. Pharmacogn. Mag..

[15] Crascì L., Lauro M.R., Puglisi G., Panico A. (2018). Natural antioxidant polyphenols on inflammation management: Anti-glycation activity vs metalloproteinases inhibition. Crit. Rev. Food Sci. Nutr..

[16] Kinger M., Kumar S., Kumar V. (2018). Some Important Dietary Polyphenolic Compounds: An Anti-inflammatory and Immunoregulatory Perspective. Mini. Rev. Med. Chem..

[17] Sharma U.K., Sharma A.K., Pandey A.K. (2016). Medicinal attributes of major phenylpropanoids present in cinnamon. BMC Complement. Altern. Med..

[18] Luo C., Xu X., Wei X., Feng W., Huang H., Liu H., Xu R., Lin J., Han L., Zhang D. (2019). Natural medicines for the treatment of fatigue: Bioactive components, pharmacology, and mechanisms. Pharmacol. Res..

[19] Singh A.K., Bishayee A., Pandey A.K. (2018). Targeting Histone Deacetylases with Natural and Synthetic Agents: An Emerging Anticancer Strategy. Nutrients.

[20] Di Lorenzo C., Colombo F., Biella S., Stockley C., Restani P. (2021). Polyphenols and Human Health: The Role of Bioavailability. Nutrients.

[21] Cortes-Martin A., Selma M.V., Tomas-Barberan F.A., Gonzalez-Sarrias A., Espin J.C. (2020). Where to Look into the Puzzle of Polyphenols and Health? The Postbiotics and Gut Microbiota Associated with Human Metabotypes. Mol. Nutr. Food Res..

[22] Tomas-Barberan F.A., Gonzalez-Sarrias A., Garcia-Villalba R., Nunez-Sanchez M.A., Selma M.V., Garcia-Conesa M.T., Espin J.C. (2017). Urolithins, the rescue of "old" metabolites to understand a "new" concept: Metabotypes as a nexus among phenolic metabolism, microbiota dysbiosis, and host health status. Mol. Nutr. Food Res..

[23] Cueva C., Silva M., Pinillos I., Bartolome B., Moreno-Arribas M.V. (2020). Interplay between Dietary Polyphenols and Oral and Gut Microbiota in the Development of Colorectal Cancer. Nutrients.

[24] Kumar Singh A., Cabral C., Kumar R., Ganguly R., Kumar Rana H., Gupta A., Rosaria Lauro M., Carbone C., Reis F., Pandey A.K. (2019). Beneficial Effects of Dietary Polyphenols on Gut Microbiota and Strategies to Improve Delivery Efficiency. Nutrients.

[25] Han Y., Xiao H. (2020). Whole Food-Based Approaches to Modulating Gut Microbiota and Associated Diseases. Annu. Rev. Food Sci. Technol..

[26] Espín J.C., González-Sarrías A., Tomás-Barberán F.A. (2017). The gut microbiota: A key factor in the therapeutic effects of (poly)phenols. Biochem. Pharmacol..

[27] Duenas M., Cueva C., Munoz-Gonzalez I., Jimenez-Giron A., Sanchez-Patan F., Santos-Buelga C., Moreno-Arribas M.V., Bartolome B. (2015). Studies on Modulation of Gut Microbiota by Wine Polyphenols: From Isolated Cultures to Omic Approaches. Antioxidants.

[28] Clifford M.N. (2004). Diet-derived phenols in plasma and tissues and their implications for health. Planta Med..

[29] Hervert D., Goñi I. (2011). Dietary Polyphenols and Human Gut Microbiota: A Review. Food Rev. Int..

[30] Saura-Calixto F., Pérez-Jiménez J., Touriño S., Serrano J., Fuguet E., Torres J.L., Goñi I. (2010). Proanthocyanidin metabolites associated with dietary fibre from *in vitro* colonic fermentation and proanthocyanidin metabolites in human plasma. Mol. Nutr. Food Res..

[31] Cortés-Martín A., García-Villalba R., González-Sarrías A., Romo-Vaquero M., Loria-Kohen V., Ramírez-de-Molina A., Tomás-Barberán F.A., Selma M.V., Espín J.C. (2018). The gut microbiota urolithin metabotypes revisited: The human metabolism of ellagic acid is mainly determined by aging. Food Funct..

[32] Zhang L., Wang Y., Li D., Ho C.T., Li J., Wan X. (2016). The absorption, distribution, metabolism and excretion of procyanidins. Food Funct..

[33] Requena T., Monagas M., Pozo-Bayón M.A., Martín-Álvarez P.J., Bartolomé B., del Campo R., Ávila M., Martínez-Cuesta M.C., Peláez C., Moreno-Arribas M.V. (2010). Perspectives of the potential implications of wine polyphenols on human oral and gut microbiota. Trends Food Sci. Technol..

[34] Sekirov I., Russell S.L., Antunes L.C., Finlay B.B. (2010). Gut microbiota in health and disease. Physiol. Rev..

[35] Eckburg P.B., Bik E.M., Bernstein C.N., Purdom E., Dethlefsen L., Sargent M., Gill S.R., Nelson K.E., Relman D.A. (2005). Diversity of the human intestinal microbial flora. Science.

[36] Gibson G.R. (1998). Dietary modulation of the human gut microflora using prebiotics. Br. J. Nutr..

[37] Salminen S., von Wright A., Morelli L., Marteau P., Brassart D., de Vos W.M., Fondén R., Saxelin M., Collins K., Mogensen G. (1998). Demonstration of safety of probiotics—A review. Int. J. Food Microbiol..

[38] Rastall R.A., Gibson G.R., Gill H.S., Guarner F., Klaenhammer T.R., Pot B., Reid G., Rowland I.R., Sanders M.E. (2005). Modulation of the microbial ecology of the human colon by probiotics, prebiotics and synbiotics to enhance human health: An overview of enabling science and potential applications. FEMS Microbiol. Ecol..

[39] Kamada N., Núñez G. (2013). Role of the gut microbiota in the development and function of lymphoid cells. J. Immunol..

[40] Krishnan S., Alden N., Lee K. (2015). Pathways and functions of gut microbiota metabolism impacting host physiology. Curr. Opin. Biotechnol..

[41] Li Y., Li J., Xu F., Liu G., Pang B., Liao N., Li H., Shi J. (2021). Gut microbiota as a potential target for developing anti-fatigue foods. Crit. Rev. Food Sci. Nutr..

[42] Yamashita M. (2020). Potential Role of Neuroactive Tryptophan Metabolites in Central Fatigue: Establishment of the Fatigue Circuit. Int. J. Tryptophan Res..

[43] Liu Z., Wu Y., Liu T., Li R., Xie M. (2017). Serotonin regulation in a rat model of exercise-induced chronic fatigue. Neuroscience.

[44] Agus A., Planchais J., Sokol H. (2018). Gut Microbiota Regulation of Tryptophan Metabolism in Health and Disease. Cell Host Microbe.

[45] Cryan J.F., Dinan T.G. (2012). Mind-altering microorganisms: The impact of the gut microbiota on brain and behaviour. Nat. Rev. Neurosci..

[46] Comai S., Bertazzo A., Brughera M., Crotti S. (2020). Tryptophan in health and disease. Adv. Clin. Chem..

[47] Rankin A., O’Donovan C., Madigan S.M., O’Sullivan O., Cotter P.D. (2017). ’Microbes in sport’—The potential role of the gut microbiota in athlete health and performance. Br. J. Sports Med..

[48] Karl J.P., Hatch A.M., Arcidiacono S.M., Pearce S.C., Pantoja-Feliciano I.G., Doherty L.A., Soares J.W. (2018). Effects of Psychological, Environmental and Physical Stressors on the Gut Microbiota. Front. Microbiol..

[49] Xiao M., Lin L., Chen H., Ge X., Huang Y., Zheng Z., Li S., Pan Y., Liu B., Zeng F. (2020). Anti-fatigue property of the oyster polypeptide fraction and its effect on gut microbiota in mice. Food Funct..

[50] Yuan X., Xu S., Huang H., Liang J., Wu Y., Li C., Yuan H., Zhao X., Lai X., Hou S. (2018). Influence of excessive exercise on immunity, metabolism, and gut microbial diversity in an overtraining mice model. Scand. J. Med. Sci. Sports.

[51] Meeusen R., Roelands B. (2018). Fatigue: Is it all neurochemistry?. Eur. J. Sport Sci..

[52] Takahashi K., Terashima H., Kohno K., Ohkohchi N. (2013). A stand-alone synbiotic treatment for the prevention of D-lactic acidosis in short bowel syndrome. Int. Surg..

[53] Shin N.R., Whon T.W., Bae J.W. (2015). Proteobacteria: Microbial signature of dysbiosis in gut microbiota. Trends Biotechnol..

[54] de Oliveira E.P., Burini R.C., Jeukendrup A. (2014). Gastrointestinal complaints during exercise: Prevalence, etiology, and nutritional recommendations. Sports Med..

[55] Donnachie E., Schneider A., Mehring M., Enck P. (2018). Incidence of irritable bowel syndrome and chronic fatigue following GI infection: A population-level study using routinely collected claims data. Gut.

[56] Konig R.S., Albrich W.C., Kahlert C.R., Bahr L.S., Lober U., Vernazza P., Scheibenbogen C., Forslund S.K. (2021). The Gut Microbiome in Myalgic Encephalomyelitis (ME)/Chronic Fatigue Syndrome (CFS). Front. Immunol..

[57] Lakhan S.E., Kirchgessner A. (2010). Gut inflammation in chronic fatigue syndrome. Nutr. Metab..

[58] Frémont M., Coomans D., Massart S., De Meirleir K. (2013). High-throughput 16S rRNA gene sequencing reveals alterations of intestinal microbiota in myalgic encephalomyelitis/chronic fatigue syndrome patients. Anaerobe.

[59] Giloteaux L., Goodrich J.K., Walters W.A., Levine S.M., Ley R.E., Hanson M.R. (2016). Reduced diversity and altered composition of the gut microbiome in individuals with myalgic encephalomyelitis/chronic fatigue syndrome. Microbiome.

[60] Shukla S.K., Cook D., Meyer J., Vernon S.D., Le T., Clevidence D., Robertson C.E., Schrodi S.J., Yale S., Frank D.N. (2015). Changes in Gut and Plasma Microbiome following Exercise Challenge in Myalgic Encephalomyelitis/Chronic Fatigue Syndrome (ME/CFS). PLoS ONE.

[61] Nacul L.C., Lacerda E.M., Campion P., Pheby D., Drachler Mde L., Leite J.C., Poland F., Howe A., Fayyaz S., Molokhia M. (2011). The functional status and well being of people with myalgic encephalomyelitis/chronic fatigue syndrome and their carers. BMC Public Health.

[62] Galland L. (2014). The gut microbiome and the brain. J. Med. Food.

[63] Maes M., Mihaylova I., Leunis J.C. (2007). Increased serum IgA and IgM against LPS of enterobacteria in chronic fatigue syndrome (CFS): Indication for the involvement of gram-negative enterobacteria in the etiology of CFS and for the presence of an increased gut-intestinal permeability. J. Affect. Disord..

[64] Sheedy J.R., Wettenhall R.E., Scanlon D., Gooley P.R., Lewis D.P., McGregor N., Stapleton D.I., Butt H.L., KL D.E.M. (2009). Increased d-lactic Acid intestinal bacteria in patients with chronic fatigue syndrome. In Vivo.

[65] Marchesi J.R., Adams D.H., Fava F., Hermes G.D., Hirschfield G.M., Hold G., Quraishi M.N., Kinross J., Smidt H., Tuohy K.M. (2016). The gut microbiota and host health: A new clinical frontier. Gut.

[66] Hsu Y.J., Chiu C.C., Li Y.P., Huang W.C., Huang Y.T., Huang C.C., Chuang H.L. (2015). Effect of intestinal microbiota on exercise performance in mice. J. Strength Cond. Res..

[67] Belkaid Y., Hand T.W. (2014). Role of the microbiota in immunity and inflammation. Cell.

[68] Lambert J.E., Myslicki J.P., Bomhof M.R., Belke D.D., Shearer J., Reimer R.A. (2015). Exercise training modifies gut microbiota in normal and diabetic mice. Appl. Physiol. Nutr. Metab..

[69] Smith P., Willemsen D., Popkes M., Metge F., Gandiwa E., Reichard M., Valenzano D.R. (2017). Regulation of life span by the gut microbiota in the short-lived African turquoise killifish. Elife.

[70] Zivkovic M., Hidalgo-Cantabrana C., Kojic M., Gueimonde M., Golic N., Ruas-Madiedo P. (2015). Capability of exopolysaccharide-producing Lactobacillus paraplantarum BGCG11 and its non-producing isogenic strain NB1, to counteract the effect of enteropathogens upon the epithelial cell line HT29-MTX. Food Res. Int..

[71] Azad M.A.K., Sarker M., Li T., Yin J. (2018). Probiotic Species in the Modulation of Gut Microbiota: An Overview. Biomed. Res. Int..

[72] Chen Y.M., Wei L., Chiu Y.S., Hsu Y.J., Tsai T.Y., Wang M.F., Huang C.C. (2016). Lactobacillus plantarum TWK10 Supplementation Improves Exercise Performance and Increases Muscle Mass in Mice. Nutrients.

[73] Jäger R., Mohr A.E., Pugh J.N. (2020). Recent advances in clinical probiotic research for sport. Curr. Opin. Clin. Nutr. Metab. Care.

[74] Flint H.J., Bayer E.A., Rincon M.T., Lamed R., White B.A. (2008). Polysaccharide utilization by gut bacteria: Potential for new insights from genomic analysis. Nat. Rev. Microbiol..

[75] Wu G.D., Chen J., Hoffmann C., Bittinger K., Chen Y.Y., Keilbaugh S.A., Bewtra M., Knights D., Walters W.A., Knight R. (2011). Linking long-term dietary patterns with gut microbial enterotypes. Science.

[76] Foster J.A., Baker G.B., Dursun S.M. (2021). The Relationship between the Gut Microbiome-Immune System-Brain Axis and Major Depressive Disorder. Front. Neurol..

[77] Zhang N., Mao X., Li R.W., Hou E., Wang Y., Xue C., Tang Q. (2017). Neoagarotetraose protects mice against intense exercise-induced fatigue damage by modulating gut microbial composition and function. Mol. Nutr. Food Res..

[78] De Preter V., Geboes K.P., Bulteel V., Vandermeulen G., Suenaert P., Rutgeerts P., Verbeke K. (2011). Kinetics of butyrate metabolism in the normal colon and in ulcerative colitis: The effects of substrate concentration and carnitine on the β-oxidation pathway. Aliment. Pharmacol. Ther..

[79] Pluznick J.L. (2017). Microbial Short-Chain Fatty Acids and Blood Pressure Regulation. Curr. Hypertens Rep..

[80] Scheiman J., Luber J.M., Chavkin T.A., MacDonald T., Tung A., Pham L.D., Wibowo M.C., Wurth R.C., Punthambaker S., Tierney B.T. (2019). Meta-omics analysis of elite athletes identifies a performance-enhancing microbe that functions via lactate metabolism. Nat. Med..

[81] Hardy H., Harris J., Lyon E., Beal J., Foey A.D. (2013). Probiotics, prebiotics and immunomodulation of gut mucosal defences: Homeostasis and immunopathology. Nutrients.

[82] Zhou K. (2017). Strategies to promote abundance of Akkermansia muciniphila, an emerging probiotics in the gut, evidence from dietary intervention studies. J. Funct. Foods.

[83] Hänninen A., Toivonen R., Pöysti S., Belzer C., Plovier H., Ouwerkerk J.P., Emani R., Cani P.D., De Vos W.M. (2018). Akkermansia muciniphila induces gut microbiota remodelling and controls islet autoimmunity in NOD mice. Gut.

[84] Lavefve L., Howard L.R., Carbonero F. (2020). Berry polyphenols metabolism and impact on human gut microbiota and health. Food Funct..

[85] Yin R., Kuo H.C., Hudlikar R., Sargsyan D., Li S., Wang L., Wu R., Kong A.N. (2019). Gut microbiota, dietary phytochemicals and benefits to human health. Curr. Pharmacol. Rep..

[86] Roopchand D.E., Carmody R.N., Kuhn P., Moskal K., Rojas-Silva P., Turnbaugh P.J., Raskin I. (2015). Dietary Polyphenols Promote Growth of the Gut Bacterium Akkermansia muciniphila and Attenuate High-Fat Diet-Induced Metabolic Syndrome. Diabetes.

[87] Fidelis M., Santos J.S., Escher G.B., Rocha R.S., Cruz A.G., Cruz T.M., Marques M.B., Nunes J.B., do Carmo M.A.V., de Almeida L.A. (2021). Polyphenols of jabuticaba [Myrciaria jaboticaba (Vell.) O.Berg] seeds incorporated in a yogurt model exert antioxidant activity and modulate gut microbiota of 1,2-dimethylhydrazine-induced colon cancer in rats. Food Chem..

[88] Dueñas M., Muñoz-González I., Cueva C., Jiménez-Girón A., Sánchez-Patán F., Santos-Buelga C., Moreno-Arribas M.V., Bartolomé B. (2015). A survey of modulation of gut microbiota by dietary polyphenols. Biomed. Res. Int..

[89] Bialonska D., Ramnani P., Kasimsetty S.G., Muntha K.R., Gibson G.R., Ferreira D. (2010). The influence of pomegranate by-product and punicalagins on selected groups of human intestinal microbiota. Int. J. Food Microbiol..

[90] Larrosa M., González-Sarrías A., Yáñez-Gascón M.J., Selma M.V., Azorín-Ortuño M., Toti S., Tomás-Barberán F., Dolara P., Espín J.C. (2010). Anti-inflammatory properties of a pomegranate extract and its metabolite urolithin-A in a colitis rat model and the effect of colon inflammation on phenolic metabolism. J. Nutr. Biochem..

[91] Anhê F.F., Roy D., Pilon G., Dudonné S., Matamoros S., Varin T.V., Garofalo C., Moine Q., Desjardins Y., Levy E. (2015). A polyphenol-rich cranberry extract protects from diet-induced obesity, insulin resistance and intestinal inflammation in association with increased Akkermansia spp. population in the gut microbiota of mice. Gut.

[92] Hidalgo M., Oruna-Concha M.J., Kolida S., Walton G.E., Kallithraka S., Spencer J.P., de Pascual-Teresa S. (2012). Metabolism of anthocyanins by human gut microflora and their influence on gut bacterial growth. J. Agric. Food Chem..

[93] Zhou L., Xie M., Yang F., Liu J. (2020). Antioxidant activity of high purity blueberry anthocyanins and the effects on human intestinal microbiota. LWT.

[94] Peng Y., Yan Y., Wan P., Dong W., Huang K., Ran L., Mi J., Lu L., Zeng X., Cao Y. (2020). Effects of long-term intake of anthocyanins from Lycium ruthenicum Murray on the organism health and gut microbiota *in vivo*. Food Res. Int..

[95] Bowey E., Adlercreutz H., Rowland I. (2003). Metabolism of isoflavones and lignans by the gut microflora: A study in germ-free and human flora associated rats. Food Chem. Toxicol..

[96] Terada A., Hara H., Nakajyo S., Ichikawa H., Hara Y., Fukai K., Kobayashi Y., Mitsuoka T. (1993). Effect of Supplements of Tea Polyphenols on the Caeeal Flora and Caeeal Metabolites of Chicks. Microb. Ecol. Health Dis..

[97] Kemperman R., Gross G., Mondot S., Possemiers S., Marzorati M., Van de Wiele T., Dore J., Vaughan E. (2013). Impact of polyphenols from black tea and red wine/grape juice on a gut model microbiome. Food Res. Int..

[98] Cheng M., Zhang X., Miao Y., Cao J., Wu Z., Weng P. (2017). The modulatory effect of (-)-epigallocatechin 3-O-(3-O-methyl) gallate (EGCG3″Me) on intestinal microbiota of high fat diet-induced obesity mice model. Food. Res. Int..

[99] Cheng M., Zhang X., Zhu J., Cheng L., Cao J., Wu Z., Weng P., Zheng X. (2018). A metagenomics approach to the intestinal microbiome structure and function in high fat diet-induced obesity mice fed with oolong tea polyphenols. Food Funct..

[100] Guo T., Song D., Cheng L., Zhang X. (2019). Interactions of tea catechins with intestinal microbiota and their implication for human health. Food Sci. Biotechnol..

[101] Zhang X., Zhu X., Sun Y., Hu B., Sun Y., Jabbar S., Zeng X. (2013). Fermentation *in vitro* of EGCG, GCG and EGCG3"Me isolated from Oolong tea by human intestinal microbiota. Food Res. Int..

[102] Bancirova M. (2010). Comparison of the antioxidant capacity and the antimicrobial activity of black and green tea. Food Res. Int..

[103] Liao Z.L., Zeng B.H., Wang W., Li G.H., Wu F., Wang L., Zhong Q.P., Wei H., Fang X. (2016). Impact of the Consumption of Tea Polyphenols on Early Atherosclerotic Lesion Formation and Intestinal Bifidobacteria in High-Fat-Fed ApoE(-/-) Mice. Front. Nutr..

[104] Jin J.S., Touyama M., Hisada T., Benno Y. (2012). Effects of green tea consumption on human fecal microbiota with special reference to Bifidobacterium species. Microbiol. Immunol..

[105] Cueva C., Sánchez-Patán F., Monagas M., Walton G.E., Gibson G.R., Martín-Álvarez P.J., Bartolomé B., Moreno-Arribas M.V. (2013). *in vitro* fermentation of grape seed flavan-3-ol fractions by human faecal microbiota: Changes in microbial groups and phenolic metabolites. FEMS Microbiol. Ecol..

[106] Sánchez-Patán F., Cueva C., Monagas M., Walton G., Gibson G., Quintanilla-López J., Lebrón-Aguilar R., Martin-Alvarez P.J., Moreno-Arribas M.V., Bartolomé B. (2012). *in vitro* Fermentation of a Red Wine Extract by Human Gut Microbiota: Changes in Microbial Groups and Formation of Phenolic Metabolites. J. Agric. Food Chem..

[107] Dolara P., Luceri C., De Filippo C., Femia A.P., Giovannelli L., Caderni G., Cecchini C., Silvi S., Orpianesi C., Cresci A. (2005). Red wine polyphenols influence carcinogenesis, intestinal microflora, oxidative damage and gene expression profiles of colonic mucosa in F344 rats. Mutat. Res..

[108] Choy Y.Y., Quifer-Rada P., Holstege D.M., Frese S.A., Calvert C.C., Mills D.A., Lamuela-Raventos R.M., Waterhouse A.L. (2014). Phenolic metabolites and substantial microbiome changes in pig feces by ingesting grape seed proanthocyanidins. Food Funct..

[109] Queipo-Ortuño M.I., Boto-Ordóñez M., Murri M., Gomez-Zumaquero J.M., Clemente-Postigo M., Estruch R., Cardona Diaz F., Andrés-Lacueva C., Tinahones F.J. (2012). Influence of red wine polyphenols and ethanol on the gut microbiota ecology and biochemical biomarkers. Am. J. Clin. Nutr..

[110] Tzounis X., Rodriguez-Mateos A., Vulevic J., Gibson G.R., Kwik-Uribe C., Spencer J.P. (2011). Prebiotic evaluation of cocoa-derived flavanols in healthy humans by using a randomized, controlled, double-blind, crossover intervention study. Am. J. Clin. Nutr..

[111] Massot-Cladera M., Pérez-Berezo T., Franch A., Castell M., Pérez-Cano F.J. (2012). Cocoa modulatory effect on rat faecal microbiota and colonic crosstalk. Arch. Biochem. Biophys..

[112] Ribeiro T.B., Costa C.M., Bonifácio - Lopes T., Silva S., Veiga M., Monforte A.R., Nunes J., Vicente A.A., Pintado M. (2021). Prebiotic effects of olive pomace powders in the gut: *in vitro* evaluation of the inhibition of adhesion of pathogens, prebiotic and antioxidant effects. Food Hydrocoll..

[113] Bao T., Li Y., Xie J., Jia Z., Chen W. (2019). Systematic evaluation of polyphenols composition and antioxidant activity of mulberry cultivars subjected to gastrointestinal digestion and gut microbiota fermentation. J. Funct. Foods.

[114] Gowd V., Xie L., Sun C., Chen W. (2020). Phenolic profile of bayberry followed by simulated gastrointestinal digestion and gut microbiota fermentation and its antioxidant potential in HepG2 cells. J. Funct. Foods.

[115] Shannon E., Conlon M., Hayes M. (2022). The Prebiotic Effect of Australian Seaweeds on Commensal Bacteria and Short Chain Fatty Acid Production in a Simulated Gut Model. Nutrients.

[116] Tajiri K., Futsukaichi Y., Kobayashi S., Yasumura S., Takahara T., Minemura M., Sugiyama T. (2018). L-Carnitine for the Treatment of Overt Hepatic Encephalopathy in Patients with Advanced Liver Cirrhosis. J. Nutr. Sci. Vitaminol..

[117] Ray Hamidie R.D., Yamada T., Ishizawa R., Saito Y., Masuda K. (2015). Curcumin treatment enhances the effect of exercise on mitochondrial biogenesis in skeletal muscle by increasing cAMP levels. Metabolism.

[118] Vamanu E., Gatea F., Sârbu I., Pelinescu D. (2019). An *in vitro* Study of the Influence of Curcuma longa Extracts on the Microbiota Modulation Process, In Patients with Hypertension. Pharmaceutics.

[119] Chen Y.M., Chiu W.C., Chiu Y.S., Li T., Sung H.C., Hsiao C.Y. (2020). Supplementation of nano-bubble curcumin extract improves gut microbiota composition and exercise performance in mice. Food Funct..

[120] Pluznick J. (2014). A novel SCFA receptor, the microbiota, and blood pressure regulation. Gut Microbes.

[121] Kimura I., Inoue D., Maeda T., Hara T., Ichimura A., Miyauchi S., Kobayashi M., Hirasawa A., Tsujimoto G. (2011). Short-chain fatty acids and ketones directly regulate sympathetic nervous system via G protein-coupled receptor 41 (GPR41). Proc. Natl. Acad. Sci. USA.

[122] Bereswill S., Muñoz M., Fischer A., Plickert R., Haag L.M., Otto B., Kühl A.A., Loddenkemper C., Göbel U.B., Heimesaat M.M. (2010). Anti-inflammatory effects of resveratrol, curcumin and simvastatin in acute small intestinal inflammation. PLoS ONE.

[123] Xue B., Xie J., Huang J., Chen L., Gao L., Ou S., Wang Y., Peng X. (2016). Plant polyphenols alter a pathway of energy metabolism by inhibiting fecal Bacteroidetes and Firmicutes *in vitro*. Food Funct..

[124] Firrman J., Liu L., Zhang L., Arango Argoty G., Wang M., Tomasula P., Kobori M., Pontious S., Xiao W. (2016). The effect of quercetin on genetic expression of the commensal gut microbes Bifidobacterium catenulatum, Enterococcus caccae and Ruminococcus gauvreauii. Anaerobe.

[125] Etxeberria U., Arias N., Boqué N., Macarulla M.T., Portillo M.P., Martínez J.A., Milagro F.I. (2015). Reshaping faecal gut microbiota composition by the intake of trans-resveratrol and quercetin in high-fat sucrose diet-fed rats. J. Nutr. Biochem..

[126] Larrosa M., Yañéz-Gascón M.J., Selma M.V., González-Sarrías A., Toti S., Cerón J.J., Tomás-Barberán F., Dolara P., Espín J.C. (2009). Effect of a low dose of dietary resveratrol on colon microbiota, inflammation and tissue damage in a DSS-induced colitis rat model. J. Agric. Food Chem..

[127] Qiao Y., Sun J., Xia S., Tang X., Shi Y., Le G. (2014). Effects of resveratrol on gut microbiota and fat storage in a mouse model with high-fat-induced obesity. Food Funct..

[128] Sung M.M., Byrne N.J., Robertson I.M., Kim T.T., Samokhvalov V., Levasseur J., Soltys C.L., Fung D., Tyreman N., Denou E. (2017). Resveratrol improves exercise performance and skeletal muscle oxidative capacity in heart failure. Am. J. Physiol. Heart Circ. Physiol..

[129] Lee H.C., Jenner A.M., Low C.S., Lee Y.K. (2006). Effect of tea phenolics and their aromatic fecal bacterial metabolites on intestinal microbiota. Res. Microbiol..

[130] Pandurangan A.K., Mohebali N., Esa N.M., Looi C.Y., Ismail S., Saadatdoust Z. (2015). Gallic acid suppresses inflammation in dextran sodium sulfate-induced colitis in mice: Possible mechanisms. Int. Immunopharmacol..

[131] Tzounis X., Vulevic J., Kuhnle G.G., George T., Leonczak J., Gibson G.R., Kwik-Uribe C., Spencer J.P. (2008). Flavanol monomer-induced changes to the human faecal microflora. Br. J. Nutr..

[132] Eom T., Ko G., Kim K.C., Kim J.S., Unno T. (2020). Dendropanax morbifera Leaf Extracts Improved Alcohol Liver Injury in Association with Changes in the Gut Microbiota of Rats. Antioxidants.

[133] da Silva-Maia J.K., Batista A.G., Correa L.C., Lima G.C., Bogusz Junior S., Maróstica Junior M.R. (2019). Aqueous extract of berry (*Plinia jaboticaba*) byproduct modulates gut microbiota and maintains the balance on antioxidant defense system in rats. J. Food Biochem..

[134] Thilakarathna W.P.D.W., Langille M.G.I., Rupasinghe H.V. (2018). Polyphenol-based prebiotics and synbiotics: Potential for cancer chemoprevention. Curr. Opin. Food Sci..

[135] Xu J., Chen H.B., Li S.L. (2017). Understanding the Molecular Mechanisms of the Interplay Between Herbal Medicines and Gut Microbiota. Med. Res. Rev..

[136] Santangelo R., Silvestrini A., Mancuso C. (2019). Ginsenosides, catechins, quercetin and gut microbiota: Current evidence of challenging interactions. Food Chem. Toxicol..

[137] Hein E.M., Rose K., van’t Slot G., Friedrich A.W., Humpf H.U. (2008). Deconjugation and degradation of flavonol glycosides by pig cecal microbiota characterized by Fluorescence in situ hybridization (FISH). J. Agric. Food Chem..

[138] Mayo B., Vázquez L., Flórez A.B. (2019). Equol: A Bacterial Metabolite from The Daidzein Isoflavone and Its Presumed Beneficial Health Effects. Nutrients.

[139] Dey P. (2019). Gut microbiota in phytopharmacology: A comprehensive overview of concepts, reciprocal interactions, biotransformations and mode of actions. Pharmacol. Res..

[140] Selma M.V., Tomás-Barberán F.A., Beltrán D., García-Villalba R., Espín J.C. (2014). Gordonibacter urolithinfaciens sp. nov., a urolithin-producing bacterium isolated from the human gut. Int. J. Syst. Evol. Microbiol..

[141] Selma M.V., Beltrán D., García-Villalba R., Espín J.C., Tomás-Barberán F.A. (2014). Description of urolithin production capacity from ellagic acid of two human intestinal Gordonibacter species. Food Funct..

[142] Beltrán D., Romo-Vaquero M., Espín J.C., Tomás-Barberán F.A., Selma M.V. (2018). *Ellagibacter isourolithinifaciens* gen. nov., sp. nov., a new member of the family *Eggerthellaceae*, isolated from human gut. Int. J. Syst. Evol. Microbiol..

[143] Jayatunga D.P.W., Hone E., Khaira H., Lunelli T., Singh H., Guillemin G.J., Fernando B., Garg M.L., Verdile G., Martins R.N. (2021). Therapeutic Potential of Mitophagy-Inducing Microflora Metabolite, Urolithin A for Alzheimer’s Disease. Nutrients.

[144] Espín J.C., Larrosa M., García-Conesa M.T., Tomás-Barberán F. (2013). Biological significance of urolithins, the gut microbial ellagic Acid-derived metabolites: The evidence so far. Evid. Based. Complement. Alternat. Med..

[145] Kawabata K., Yoshioka Y., Terao J. (2019). Role of Intestinal Microbiota in the Bioavailability and Physiological Functions of Dietary Polyphenols. Molecules.

[146] Ryu D., Mouchiroud L., Andreux P.A., Katsyuba E., Moullan N., Nicolet-Dit-Félix A.A., Williams E.G., Jha P., Lo Sasso G., Huzard D. (2016). Urolithin A induces mitophagy and prolongs lifespan in C. elegans and increases muscle function in rodents. Nat. Med..

[147] Luan P., D’Amico D., Andreux P.A., Laurila P.P., Wohlwend M., Li H., Imamura de Lima T., Place N., Rinsch C., Zanou N. (2021). Urolithin A improves muscle function by inducing mitophagy in muscular dystrophy. Sci. Transl. Med..

[148] Zhao C., Sakaguchi T., Fujita K., Ito H., Nishida N., Nagatomo A., Tanaka-Azuma Y., Katakura Y. (2016). Pomegranate-Derived Polyphenols Reduce Reactive Oxygen Species Production via SIRT3-Mediated SOD2 Activation. Oxid. Med. Cell Longev..

[149] Ghosh N., Das A., Biswas N., Gnyawali S., Singh K., Gorain M., Polcyn C., Khanna S., Roy S., Sen C.K. (2020). Urolithin A augments angiogenic pathways in skeletal muscle by bolstering NAD(+) and SIRT1. Sci. Rep..

[150] Sebastián D., Sorianello E., Segalés J., Irazoki A., Ruiz-Bonilla V., Sala D., Planet E., Berenguer-Llergo A., Muñoz J.P., Sánchez-Feutrie M. (2016). Mfn2 deficiency links age-related sarcopenia and impaired autophagy to activation of an adaptive mitophagy pathway. EMBO J..

[151] Andreux P.A., Blanco-Bose W., Ryu D., Burdet F., Ibberson M., Aebischer P., Auwerx J., Singh A., Rinsch C. (2019). The mitophagy activator urolithin A is safe and induces a molecular signature of improved mitochondrial and cellular health in humans. Nat. Metab..

[152] Palikaras K., Daskalaki I., Markaki M., Tavernarakis N. (2017). Mitophagy and age-related pathologies: Development of new therapeutics by targeting mitochondrial turnover. Pharmacol. Ther..

[153] D’Amico D., Andreux P.A., Valdés P., Singh A., Rinsch C., Auwerx J. (2021). Impact of the Natural Compound Urolithin A on Health, Disease, and Aging. Trends. Mol. Med..

[154] Tomás-Barberán F.A., García-Villalba R., González-Sarrías A., Selma M.V., Espín J.C. (2014). Ellagic acid metabolism by human gut microbiota: Consistent observation of three urolithin phenotypes in intervention trials, independent of food source, age, and health status. J. Agric. Food Chem..

[155] Selma M.V., Romo-Vaquero M., García-Villalba R., González-Sarrías A., Tomás-Barberán F.A., Espín J.C. (2016). The human gut microbial ecology associated with overweight and obesity determines ellagic acid metabolism. Food Funct..

[156] Romo-Vaquero M., Cortés-Martín A., Loria-Kohen V., Ramírez-de-Molina A., García-Mantrana I., Collado M.C., Espín J.C., Selma M.V. (2019). Deciphering the Human Gut Microbiome of Urolithin Metabotypes: Association with Enterotypes and Potential Cardiometabolic Health Implications. Mol. Nutr. Food Res..

[157] Li Z., Henning S.M., Lee R.P., Lu Q.Y., Summanen P.H., Thames G., Corbett K., Downes J., Tseng C.H., Finegold S.M. (2015). Pomegranate extract induces ellagitannin metabolite formation and changes stool microbiota in healthy volunteers. Food Funct..

